# Commentary: Transient Postural Vestibulo-Cerebellar Syndrome in Three Dogs With Presumed Cerebellar Hypoplasia

**DOI:** 10.3389/fvets.2021.613521

**Published:** 2021-02-19

**Authors:** Shinji Tamura

**Affiliations:** Tamura Animal Clinic, Hiroshima, Japan

**Keywords:** positioning head tilt, dog, cerebellar, nodules, ventral uvula

## Introduction

The use of the technical term “positioning head tilt” in a recently published article entitled “Transient Postural Vestibulo-Cerebellar Syndrome in Three Dogs with Presumed Cerebellar Hypoplasia” by Prikryl et al. seems incorrect. The authors appear to have overlooked a slight head tilt in the Case 1 movie. The correct meaning of positioning head tilt is discussed in this general commentary article.

## What is “Positioning Head Tilt?”

Positioning head tilt is a neurological sign originally described in 2016 in three dogs with presumptive cerebellar nodulus and ventral uvula hypoplasia. Dogs with positioning head tilt can turn freely in any direction. The head is in a level position when static or when the dog walks in a straight line. However, the head tilts to the opposite side when the dog turns its head (1). The cerebellum maintains the position of the head unconsciously. Signaling by the vestibular system is inputted into the vestibulocerebellum (the vermis, the fastigial nucleus, and the flocculi) upon detection of head movement. The actual head position is detected by proprioceptive receptors (muscle spindles) within the oblique and rectus capitis muscles. The signal is inputted into the spinocerebellum (the paravermian cortex and the interposed nucleus) through the spinocuneocerebellar tract (2–8). ‘Vestibular nuclear projections to the spinal cord are situated in the vestibulospinal tracts. The fibers terminate on interneurons that facilitate ipsilateral flexors and decussate to inhibit contralateral extensor muscle activity. The vestibulospinal tracts facilitate spinal reflexes, especially those involved in maintaining posture and the antigravity/extensor muscles. Turning the head to the left causes a shift in the distribution of body mass to the left. This shift in mass is supported by increased extension on the left side, which is reflexively induced by both myotatic and vestibular reflexes. This also reduces extension on the right side, and thereby minimizes weight transfer to the left’ (1). The cerebellar nodulus and ventral uvula (NU) coordinates this system by inhibition of stimulation of vestibular nuclei in order to maintain a level head position in response to head movement (1). This inhibition is absent in animals with NU dysfunction, and positioning head tilt occurs.

## Discussion

Positioning head tilt is not a vestibular deficit related to posture, as explained in the Introduction. Rather, it describes a vestibular deficit related to head movement (in other words, “postural changing”). The previously reported observation is not “positional head tilt,” as mentioned in the third paragraph of the Discussion section, but “positioning head tilt” (1). “Positional” is related to posture and “positioning” to postural changes. They have completely different meanings like “positional nystagmus” and “positioning nystagmus” in human medicine (9). Since various vestibular signs elicited by the postural changes of the head are discussed in the original article, for the same reason, “positioning vestibular-cerebellar syndrome” is a more accurate description of the condition than the “postural vestibular-cerebellar syndrome” in the title.

Positioning head tilt was observed especially in the first half of the Case 1 supplemental movie (https://www.frontiersin.org/articles/10.3389/fvets.2020.00453/full#supplementary-material); however, the authors did not observe it. Positioning head tilt can be easily overlooked, especially in animals with only mild tilting. Long-time observation of free walking is necessary to evaluate positioning head tilt.

## Author Contributions

The author confirms being the sole contributor of this work and has approved it for publication.

## Conflict of Interest

The author declares that the research was conducted in the absence of any commercial or financial relationships that could be construed as a potential conflict of interest.
